# The Fears of the Rich, the Needs of the Poor: My Years at the CDC

**DOI:** 10.3201/eid2410.180687

**Published:** 2018-10

**Authors:** Loren Robinson, Cjloe Vinoya-Chung

**Affiliations:** Commonwealth of Pennsylvania—Health, Health Promotion and Disease Prevention, West Harrisburg, Pennsylvania, USA

**Keywords:** William Foege, CDC, EIS, Fears of the rich, needs of the poor: my years at the CDC, HIV/AIDS and other retroviruses, Infectious diseases, Smallpox

To say that William Foege’s life has been inspirational is an understatement. It has encompassed literal life-and-death adventures, often intertwined with efforts to prevent and control some of the most destructive infectious diseases of the past half century. By recounting his time as an officer in the Epidemic Intelligence Service, his public health experiences in war zones, and his service as director of the Centers for Disease Control, Dr. Foege provides a front-row seat from which to watch the field of public health progress. 

*The Fears of the Rich, the Needs of the Poor: My Years at the CDC* (Figure) is well organized chronologically, with contextual information clearly documented. Foege recounts many momentous CDC adventures and introduces the reader to numerous CDC contemporaries. However, although the title may be captivating, it does not reflect the focus of the book well. Many of Foege’s assignments involved underserved and poverty-stricken populations, and he occasionally touches on the effects of wealth and class on the health of these populations, but the book is primarily about his experiences at CDC. That being said, in stating his “three essentials for good public health programs,” Foege does say: “The first is the conviction that the basis for public health is to achieve health equity; therefore, the bottom line is social justice in health.” 

**Figure:**
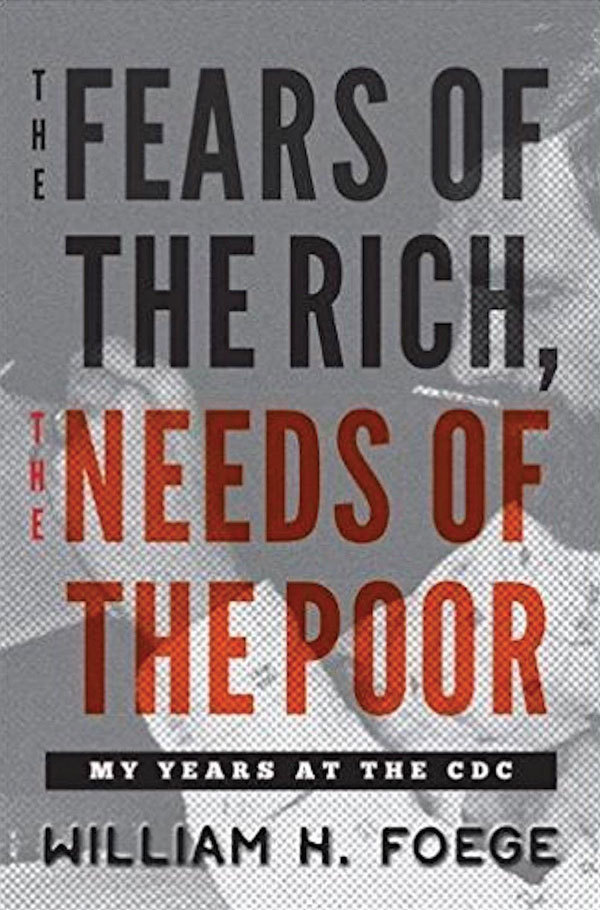
The Fears of the Rich, the Needs of the Poor: My Years at the CDC.

This book was written with a public health audience in mind but would be a fascinating read for anyone with an interest in public health. Foege’s distinct colloquial voice and dry humor personalize the book, bringing his work to life. Foege provides a first-hand account of the recent history of humanity’s struggles with infectious diseases across the world, including progress made in the field of public health over the decades of his career. By sharing real stories of infectious diseases that devastated populations and how Foege and his colleagues grew into the leaders that helped bring these epidemics under control, the book provides guidance and inspiration for current and future public health workforces. Although public health can be a thankless profession, through this memoir Foege reminds us how indispensable the field is for our world’s future. 

